# Bioinformatics analysis of lncRNA and mRNA differentially expressed in patients with cervical cancer

**DOI:** 10.3389/fbinf.2025.1605681

**Published:** 2025-08-01

**Authors:** Xiaohua An, Xiaoxue Huang, Qiujie Yu, Yiyue Tang, Yan Wang, Huasu Chen, Yafei Zhang, Qianhao Huang, Yudi Rao, Guomei Hu, He Zha

**Affiliations:** ^1^ Department of Laboratory Medicine, The Third Affiliated Hospital of Zunyi Medical University (The First People’s Hospital of Zunyi), Zunyi, Guizhou, China; ^2^ Scientific Research Center, The Third Affiliated Hospital of Zunyi Medical University (The First People’s Hospital of Zunyi), Zunyi, Guizhou, China; ^3^ Department of Cardiovascular Medicine, The Third Affiliated Hospital of Zunyi Medical University (The First People’s Hospital of Zunyi), Zunyi, Guizhou, China; ^4^ Pathology Department, The Third Affiliated Hospital of Zunyi Medical University (The First People’s Hospital of Zunyi), Zunyi, Guizhou, China

**Keywords:** cervical cancer, lncRNA, mRNA, HPV16 E6/E7, high-throughput sequencing

## Abstract

To verify the expression profile of long non-coding RNAs (lncRNAs) and mRNAs in cervical cancer, identify their clinical significance in HPV16-associated cervical cancer, and annotate the biological function of mRNAs. Three pairs of cancerous and paracancer tissues were selected in cervical squamous cell carcinoma (IB2 stage), high-throughput sequencing was utilized to determine the expression levels of lncRNAs and mRNAs. The detection results were validated by GEPIA database analysis and RT-qPCR. Functional annotations of differential mRNAs were conducted through Gene Ontology (GO), Kyoto Encyclopedia of Genes and Genomes (KEGG) pathway enrichment analysis, and protein-protein interaction (PPI) network states. Furthermore, the association between antisense lncRNA and mRNA in cervical cancer was analyzed to predict the biological functions of lncRNA. Finally, recombinant lentivirus CV224-HPV16 E6/E7 was transfected into HcerEpic to establish a stable cell line with overexpressed HPV16 E6/E7, then differential lncRNAs were detected by RT-qPCR. Compared to paracancerous tissues, there were 3,608 lncRNAs significantly upregulated and 4,383 lncRNAs significantly downregulated in cervical cancer tissues (Fold change >2 and *P* < 0.05). Additionally, 3,666 mRNAs were significantly upregulated, while 2,220 mRNAs were significantly downregulated (Fold change >2 and *P* < 0.05). GO/KEGG enrichment analysis showed that differentially expressed mRNA played a significant role in cell cycle and cell senescence, and was related to signal pathways such as cAMP and MAPK, forming a complex network among the proteins encoded by these mRNAs. Further analysis indicated that the 20 antisense lncRNAs with the most remarkable differences might exert biological functions by influencing their corresponding mRNAs. The results of RT-qPCR revealed that CDKN2B-AS1, HAGLROS and GATA6-AS1 were potentially regulated by HPV16 E6/E7, which were in accordance with those obtained from chip detection. In this study, differentially expressed lncRNAs associated with HPV16 infection were screened and explored their transcriptional molecular functions and biological pathways, providing a molecular basis for predicting diagnostic markers of cervical cancer.

## 1 Introduction

Cervical cancer is the fourth most common malignant tumor in women worldwide in terms of both incidence and fatality (Sung et al., 2021). Globally, there were about 604,127 new cervical cancer diagnoses in 2020, and 341,831 fatalities (Sung et al., 2021), which seriously threatened women’s health. Currently, cervical cancer can be treated through diverse approachcng surgery (Greggi et al., 2020), chemotherapy (Crafton and Salani, 2016), radiotherapy (Tan et al., 2019), and immunotherapy (Ferrall et al., 2021; Feng et al., 2020). However, the 5-year survival rate was only 59.8% (Zeng et al., 2018), signifying a substantial challenge in boosting the survival rates of individuals diagnosed with cervical cancer.

Cervical cancer development is closely associated with ongoing infection with high-risk human papillomaviruses (HPV). Studies have shown that the most predominant oncogenic HPV types are 16 (57%) and 18 (16%) (Crosbie et al., 2013). The E6 and E7 proteins of these high-risk HPV types possess oncogenic characteristics. However, not all patients infected with HPV will eventually develop cervical cancer, as the progression of the disease is regulated by numerous factors. The specific mechanisms of these factors are still not fully understood (Okunade, 2020). Therefore, further research on the pathogenesis of cervical cancer, together with the discovery of novel treatment targets and sensitive and specific diagnostic markers, has significant clinical value for improving the efficiency of cervical cancer diagnosis and treatment.

Long noncoding RNAs (lncRNAs) are transcripts that are more than 200 nucleotides long (Chen et al., 2022). They have the ability to control how genes that code for proteins are expressed by exerting an influence on the transcription, post-transcriptional and translation modifications of mRNAs (Quinn and Chang, 2016; Choi et al., 2019), thereby leading to tumorigenesis and progression (Bai et al., 2023; Wu et al., 2022). Depending on their position in relation to protein-coding genes, lncRNA can be classified as sense, antisense, intron, intergenic, and bidirectional (St Laurent et al., 2015). Antisense lncRNAs constitute a relatively well-studied category. More than 30% of human annotated transcripts have corresponding antisense lncRNA, which regulates the corresponding justice mRNA through various mechanisms (Faghihi and Wahlestedt, 2009).

In order to examine the impact of differential lncRNA on the tissues of cervical cancer, this study selected cancer tissues and adjacent non-cancerous tissues from three pairs of HPV16-positive cervical squamous carcinoma patients, all clinically diagnosed at stage IB2. Arraystar LncRNA and mRNA expression profiling microarrays were utilized via high-throughput sequencing technology. Bioinformatics analyses were carried out to identify lncRNAs and mRNAs, and to predict relevant downstream regulatory signaling pathways and key genes. For mRNAs, GO functional analysis, KEGG pathway enrichment analysis, and PPI assessments were used. At the cellular level, the characteristics of these lncRNAs, the signaling pathways they participate in, and their relationship to HPV16 infection were all examined using RT-qPCR validation. This study provides theoretical support for bioinformatics in outcome prediction and contributes to the diagnosis of cervical cancer.

## 2 Materials and methods

### 2.1 Materials

#### 2.1.1 Tissue specimens and cell lines

Cancer tissues and adjacent paracancerous tissues from three patients diagnosed with HPV16-positive cervical squamous carcinoma at stage IB2, from June 2021 to February 2022, were selected from the First People’s Hospital of Zunyi City. Notably, none of the patients had received preoperative radiotherapy or chemotherapy. Subsequently, the samples were sent to Shanghai Kangcheng Bioengineering Co., Ltd. for labeling and High-throughput sequencing. The hospital’s ethics committee approved this study, and each patient signed an informed consent form.

The Chinese Academy of Sciences’ Cell Bank provided the human normal cervical epithelial cells (HcerEpic) and cervical cancer cell lines (HeLa, SiHa, and C33A) used in this investigation.

#### 2.1.2 Main reagents

Beijing Solabao Technology Co. supplied the 2×SYBR Green PCR mix kit, while Gibco, USA, supplied the trypsin and DMEM high glucose medium. We purchased fetal bovine serum (FBS) from Israel Biotech; Trizol reagent was acquired from Invitrogen, USA. Chloroform was obtained from Chengdu Kelong Chemical Reagent Factory, China; Purchase PrimeScript™ RT Reagent Kit from TaKaRa, Japan; PCR primers were synthesized by Bioengineering (Shanghai) Co. Recombinant lentivirus CV224-HPV16 E6/E7 was constructed and synthesized by Shanghai Jikai Gene Science Co. The selection, probe design, image acquisition, and data analysis of the Arraystar Human LncRNA Microarray V5.0 were conducted by Shanghai Kangcheng Bioengineering Co., Ltd.

### 2.2 Methods

#### 2.2.1 Total RNA extraction and quality control

Total RNA was extracted from cervical cancer tissues and paracancerous tissues of three cases in accordance with the instructions accompanying the Trizol reagent. The paracancerous tissues served as the control group, whereas the cancer tissues formed the experimental group. Subsequently, The RNeasy Mini Kit (Qiagen) was then used to purify the RNA samples. The Agilent 2100 Bioanalyzer or conventional denatured gel electrophoresis were used to evaluate the integrity of the RNA.

#### 2.2.2 Microarray hybridization analysis

The Human LncRNA Microarray V5.0 has been carefully engineered to identify a wide range of protein-coding transcripts and 39,317 lncRNAs. The lncRNAs encompassed in this microarray have been judiciously selected from several authoritative public transcriptome databases, including FANTOM5 CAT (v1), GENECODE (v29), RefSeq, and NONCODE (v5). During the hybridization process, a random priming method was employed. Following purification with the RNeasy Mini Kit (Qiagen), the labeled cRNAs were assessed for activity and concentration using a NanoDrop ND-1000. Thereafter, microarray hybridization was performed, followed by washing, fixing, and scanning on a hybridization chip using the Agilent DNA Microarray Scanner (part number G2505C).

#### 2.2.3 Construction of differential expression profiles of lncRNA and mRNA

We used Agilent Feature Extraction software (v11.0.1.1) in this investigation to acquire mRNA and lncRNA differential expression profiles. Both raw data and microarray plots were produced using this software. Next, the raw data was processed and quantile normalized using GeneSpring GX v12.1 software (Agilent Technologies). After screening for high-quality probes, further analysis was conducted. The differential lncRNAs or mRNAs between the two sample groups was determined by screening for differential expression profiles with a significance threshold of *P* < 0.05 and a fold change >2.0.

#### 2.2.4 GO and KEGG enrichment analysis of differentially expressed mRNAs

By significantly enriching differential mRNAs based on GO terms, the analysis encompassed the Biological Process (BP), Cellular Component (CC), and Molecular Function (MF) categories to elucidate the primary biological functions of differential mRNAs and their roles in various biological processes. A smaller *P* value (*P* < 0.05) indicates a more significant GO term. Additionally, KEGG analysis was conducted to predict the signaling pathways in which differentially expressed lncRNAs may be involved. A smaller *P* value (*P* < 0.05) also signifies a more significant KEGG term.

#### 2.2.5 Analysis of interactions between mRNA-encoded proteins

Select the top 200 mRNA datasets with the most significant differences and import them into the STRING database (https://cn.string-db.org). In the display options, select ‘Hide isolated nodes in network’ and then construct a network diagram to illustrate the interactions among proteins encoded by mRNA.

#### 2.2.6 GEPIA and KM-Plotter database analysis

The expression of predicted lncRNAs in cervical cancer was analyzed via the GEPIA database (http://gepia2021.cancerpku.cn) and the KM-Plotter database (https://www.kmplot.com/analysis/) to evaluate the prognostic implications of lncRNAs in cervical cancer patients.

#### 2.2.7 RT-qPCR

RNA was extracted from cells and tissues by Trizol lysis. Subsequently, reverse transcription of cDNA was conducted. A 20 µL reaction system was prepared by using a 2× SYBR Green PCR Mix kit. Forward and reverse primers were configured according to the manufacturer’s instructions. After pre-denaturation at 95°C for 5 min (1 cycle), denaturation at 95°C for 10 s, annealing at 60°C for 30 s, extension at 72°C for 30 s (40 cycles), and a final hold at 72°C for 5 min, the reaction program was stored at 4°C for amplification. Using GAPDH as an internal reference, the 2^−ΔΔCT^ technique was used to examine the target genes. In Table 1, the gene primer sequences are displayed.

**TABLE 1:** Primer used for RT-qPCR.

Gene symbol	Sequence	Fragment size (bp)
CDKN2B-AS1	forward 5′-ATTTTATTCCTGGCTCCCCTCGTC-3′ reverse 5′-TGGCGGATAGAGCAATGAGATGAC-3′	110
DICER1-AS1	forward 5′- CGCCCTTCACTGCCTCTCTTC-3′ reverse 5′- TGCTCTGGCTGTGTCATCCTTAG-3′	122
HAGLROS	forward 5′- TGTCACCCTTAAATACCGCTCT-3′ reverse 5′- CTTCCTCCCACACAAATACTCC-3′	153
GATA6-AS1	forward 5′- TTCTGGGAGTCGCGCATT-3′ reverse 5′- GTGGCCGCATTTGGAAAA -3′	121
MIR205HG	forward 5′- GTGCTTTATATAGGAAAGGACCAAC-3′ reverse 5′- CCATGCCTCCTGAACTTCACT -3′	108
GAPDH	forward 5′- GGAGCGAGATCCCTCCAAAAT-3′ reverse 5′- GGCTGTTGTCATACTTCTCATGG -3′	193

#### 2.2.8 Cell transfection

HcerEpic cells were diluted to 5 × 10^4^ cells/mL of cell suspension, and then incubated in a 37°C incubator for 24 h. Once the cell density reached 70%, the lentivirus CV224-HPV16 E6/E7 along with co-transfection reagents constructed and packaged by Shanghai GeneChem Co., Ltd. were used for HcerEpic. The medium was swapped out for new complete media 24 h following transfection. After 48–72 h, the cells were examined for infection using an inverted fluorescence microscope, and stable transfected cell strains were selected using 2 μg/mL puromycin for subsequent experiments.

#### 2.2.9 Statistical analysis

Software such as SPSS 29.0 and GraphPad Prism 6 were used to statistically evaluate the data outputs. Mean ± standard deviation (‾*x ± s*) was used to convey measurement data that fit a normal distribution, while n (%) was used to represent count data. A paired *t*-test was utilized for intra-group comparisons, while an independent samples *t*-test was utilized for comparisons between the two groups. Non-parametric tests were utilized for data that did not fit a normal distribution; for between-group comparisons of count data, the *χ* ^*2*^ test or Fisher’s exact test was employed. The threshold for statistical significance was set at *P* < 0.05.

## 3 Results

### 3.1 LncRNA and mRNA differential expression analysis

**FIGURE 1:**
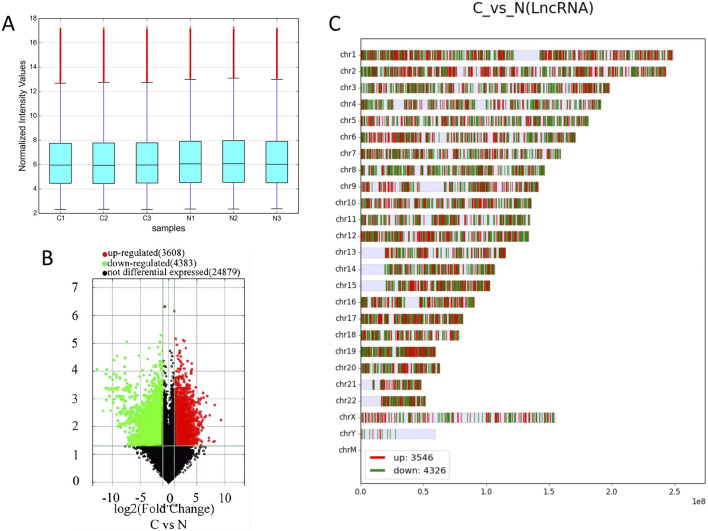
Differentially expressed lncRNAs in cervical cancer patients. **(A)** Box plot of the dispersion of the six sets of data for lncRNAs. **(B)** Volcano plot of lncRNAs with differential expression, with 2-fold upregulated lncRNAs in red and 2-fold downregulated lncRNAs in green. **(C)** Distribution of lncRNAs in chromosomes.

Box plots comparing the experimental group with the control group showed that the dispersion of lncRNAs and mRNAs data among the six groups was largely consistent (Figure 1A; Supplementary Figure S1A). A total of 3608 of the 7991 lncRNAs that were found to be differentially expressed in the experimental group as opposed to the control group were upregulated, whereas 4,383 were downregulated. Furthermore, the experimental group had 5,886 differentially expressed mRNAs, of which 3,666 were upregulated and 2,220 were downregulated. Volcano plots were generated for the differentially expressed lncRNAs, effectively depicting their distribution, fold change in expression, and significance of the results. In Figure 1B; Supplementary Figure S1B, red dots represent differentially upregulated lncRNAs and mRNAs, green dots indicate differentially downregulated lncRNAs and mRNAs, and gray dots signify lncRNAs and mRNAs with no significant differential expression. The 20 lncRNAs and mRNAs with the most substantial differences in both upregulation and downregulation are detailed in Table 2 and Supplementary Table S1. Furthermore, the differentially expressed lncRNAs and mRNAs were distributed across chromosomes 1–22, as well as the X and Y chromosomes (Figure 1C; Supplementary Figure S1C). In addition, we also analyzed the 10 mRNA that were differentially upregulated and the 10 mRNA that were downregulated, as shown in Table 3.

**TABLE 2:** Most substantial differencially expressed lncRNAs in cervical cancer patients.

Upregulated lncRNAs		Downregulated lncRNAs	
lncRNA	Fold-change	lncRNA	Fold-change
CATG00000027321.1	636.9061239	G047911	6937.990902
G089593	292.4648972	HOXB-AS4	2275.910853
AL049555.1	287.5567314	CATG00000038938.1	2072.279809
CATG00000092533.1	159.8965499	AC099560.1	1018.316089
MIR205HG	146.3361637	XLOC_013557	976.8543315
LOC100130899	115.1445194	LINC00470	632.3659016
AC024587.2	99.5060611	AL139260.1	543.8341357
LINC02560	84.4043491	AC007383.2	530.9284105
CDKN2B-AS1	70.7083456	ELMO1	445.1438046
LINC01956	68.5309097	AC104964.3	428.5326448
EPHA1	62.1165931	G064089	425.7516074
AC074050.4	61.8415139	CATG00000022496.1	421.5776868
LINC00511	61.1640486	AC074389.2	413.4464294
AL021807.1	58.4494212	GATA6-AS1	399.5837675
AC007848.1	57.0298711	RASAL2-AS1	394.4119172
AL512413.1	54.9253505	LINC00958	275.103217
AC007996.1	54.0569759	AC079779.3	235.835594
KNL1	50.5805458	FOXD2-AS1	234.5802375
AL445524.1	50.0969002	AC105760.2	189.5778892
HAGLROS	48.7441743	DICER1-AS1	180.2364925

**TABLE 3:** Co-differential expression profiles for lncRNA and mRNA.

lncRNA	mRNA	Protein naming	Fold change -lncRNA	Fold change - mRNA	Expression regulation-mRNA
ZNRF3-AS1	ZNRF3	Zinc and ring finger 3	2303.751676	3.0187431	Up
G076835	TMEM243	Transmembrane protein 243	1220.575566	4.4602035	Down
AL139260.1	MYCBP	MYC binding protein	543.8341357	4.5640623	Down
AL357835.1	TNFRSF8	TNF receptor superfamily member 8	388.2729254	2.0199254	Down
AC105760.2	COPS8	COP9 signalosome subunit 8	189.5778892	2.0967095	Up
DICER1-AS1	DICER1	Dicer 1, ribonuclease III	180.2364925	3.8189894	Up
TSPOAP1-AS1	SUPT4H1	SPT4 homolog, DSIF elongation factor subunit	172.3521916	2.6175141	Up
CATG00000078061.1	PMF1-BGLAP	PMF1-BGLAP readthrough	123.6459921	2.2795893	Up
ADAMTS9-AS1	ADAMTS9	ADAM metallopeptidase with thrombospondin type 1 motif 9	120.0295931	5.0983664	Down
AC092614.1	MYO1B	Myosin IB	88.2336666	2.3434914	Up
LOC613266	MACROD2	MACRO domain containing 2	81.3197909	3.1545941	Up
CDKN2B-AS1	CDKN2B	Cyclin dependent kinase inhibitor 2B	70.7083456	102.4963838	Up
AC018529.2	MBP	Myelin basic protein	52.3701186	2.0205056	Up
RNF219-AS1	RNF219	Ring finger protein 219	52.0876677	8.4740315	Up
AL356134.1	SLC28A3	solute carrier family 28 member 3	46.4002541	182.7079416	Up
PGM5-AS1	PGM5	phosphoglucomutase 5	45.7619376	32.1046322	Down
AC120498.10	TPSG1	Tryptase gamma 1	45.6495215	10.7907811	Down
SRD5A3-AS1	SRD5A3	Steroid 5 alpha-reductase 3	40.8791889	4.0924185	up
FLJ13224	SINHCAF	SIN3-HDAC complex associated factor	40.449987	8.1963633	up
AC006449.3	MLLT6	MLLT6, PHD finger containing	39.1806764	3.6028635	up

### 3.2 GO/KEGG pathway enrichment analysis of differential mRNAs

Differentially expressed mRNAs were subjected to GO analysis. The top 10 GO terms with the greatest enrichment were displayed for each GO categorization after upregulated and downregulated differential mRNAs were chosen independently.

**FIGURE 2:**
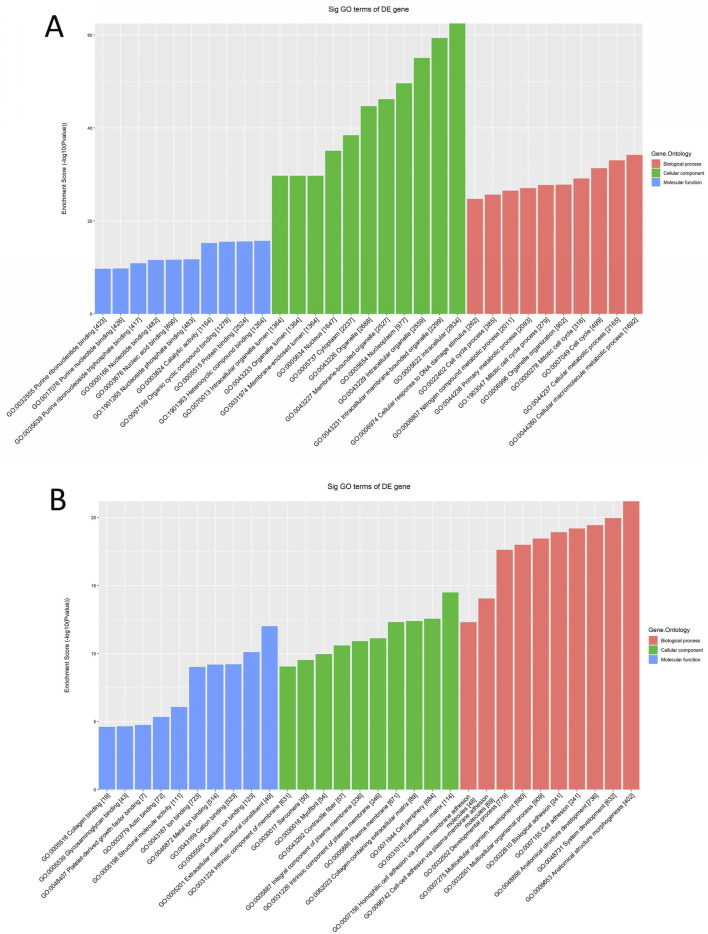
GO enrichment analysis of differentially expressed mRNAs in cervical cancer patients. **(A)** GO function annotation plot for expression of upregulated mRNAs, the horizontal axis indicates GO enrichment entries, the vertical axis indicates the number of genes, and a larger enrichment score indicates a greater degree of enrichment. **(B)** GO function annotation plot for expression of downregulated mRNAs.

The results revealed that significantly upregulated mRNAs enriched for BP were mainly involved in cellular molecular metabolic processes (GO:0044260), the cell cycle (GO:0007049), and organelle organization (GO:0006996). Enriched Cellular Components (CC) were predominantly located in the cytoplasm, linked to organelles and lumen, and involved in growth and developmental processes. Additionally, the MF observed included catalytically active nucleic acid binding, nucleotide binding, and protein binding, as illustrated in Figure 2A. On the contrary, significantly downregulated mRNA-enriched BP primarily related to multicellular biological processes (GO:0032501) and might possess bioadhesive functions (GO:0022610). The CC included myofibrils, the extracellular matrix, and the plasma membrane, while the MF encompassed protein binding and metal ion binding, among others, as shown in Figure 2B.

**FIGURE 3:**
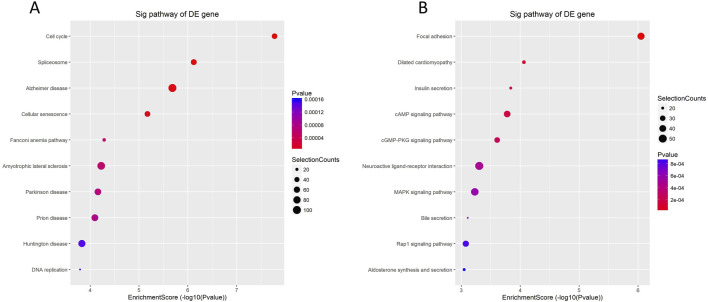
KEGG enrichment analysis of differentially expressed mRNA in cervical cancer patients. **(A)** Analysis of enrichment Bubble plot of KEGG signaling pathway expressing upregulated mRNAs: the size of the bubble indicates the number of differentially expressed genes enriched in this pathway, the colors of the bubbles represent the various P-values, and the horizontal axis of the plot shows the enrichment score and the vertical axis the pathway name. The larger the enrichment fraction, the higher the enrichment degree. **(B)** Enrichment analysis bubble plot of KEGG signaling pathway expressing downregulated mRNAs.

According to the enrichment analysis results of KEGG pathway, enrichment was evaluated through GO ID enrichment scores, *P*-values, and the quantity of mRNA target genes associated with every route. Subsequently, the top 10 KEGG pathways with the greatest enrichment were chosen to be shown. The findings showed that the significantly upregulated mRNAs were primarily engaged in the cell cycle’s control, spliceosome function, and cellular senescence, with associations to diseases such as Alzheimer’s disease, the Fanconi anemia pathway, and Parkinson’s syndrome (Figure 3A). Conversely, the significantly downregulated mRNAs were mainly linked to the secretion of substances such as insulin and bile, and were associated with signaling pathways including cAMP and MAPK, which may regulate processes such as neuroactive ligand-receptor interactions (Figure 3B).

### 3.3 Analysis of interactions between proteins encoded by differential mRNAs

**FIGURE 4:**
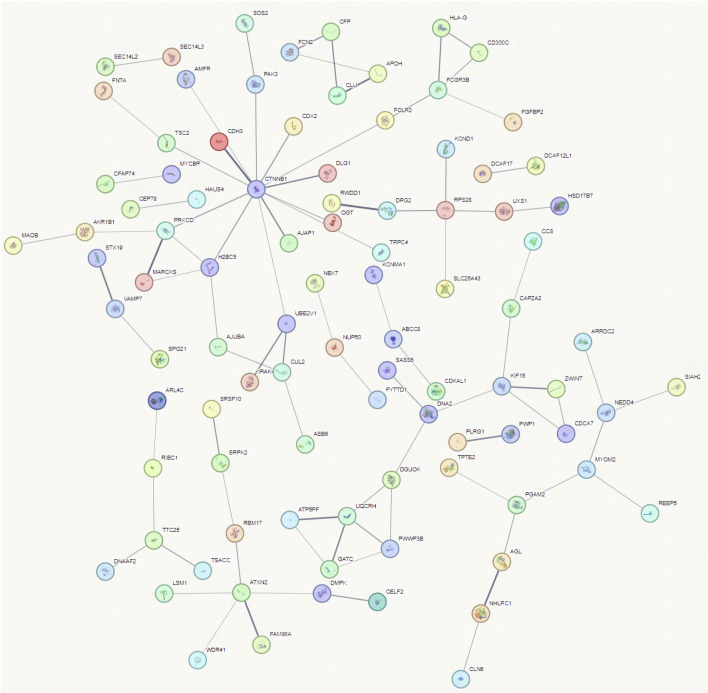
Interaction analysis between proteins encoded by differentially expressed mRNAs.

To use the STRING database to analyze protein interactions, the 100 most significantly upregulated and 100 most significantly downregulated mRNAs were chosen. The results showed that the proteins with the strongest interactions included CTNNB1, ATXN2, PRKCD, FCGR3B, UQCRH, RPS28, and KIF15 (Figure 4). CTNNB1 encodes the β-catenin protein, which is crucial to cell adhesion and signaling (Costigan et al., 2020). An RNA-binding protein called ATXN2 controls the formation of stress granules and has been linked to the etiology of a number of neurodegenerative illnesses (Akçimen et al., 2021). The protein encoded by PRKCD (Liu et al., 2020) is involved in a number of biological functions, including the negative regulation of the insulin receptor signaling cascade and the assembly of cellular components. This analysis highlights the biological significance of mRNAs in cervical cancer tissues compared to paracancerous tissues, providing theoretical support for subsequent experiments aimed at studying these targets.

### 3.4 Co-differential expression profiles and lncRNA function prediction for LncRNA and mRNA in cervical cancer patients

In studies on lncRNAs, antisense lncRNAs regulate their corresponding mRNAs through multiple mechanisms to get biological functions. And we analyzed the 20 antisense lncRNAs with the most significant differential expression and performed a co-expression analysis with the differentially expressed mRNAs to infer the functions of these lncRNAs (Table 3).

### 3.5 Validation of differentially expressed lncRNAs

#### 3.5.1 GEPIA predicts lncRNA expression in cervical cancer

**FIGURE 5:**
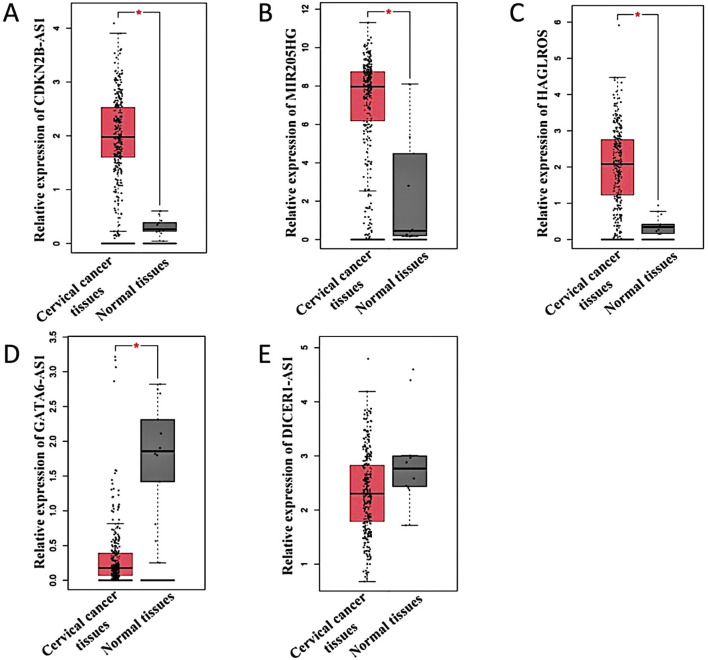
Differential expression of lncRNAs in cervical cancer. **(A–E)** Indicates the expression of GEPIA database predicted lncRNA CDKN2B-AS1, MIR205HG, HAGLROS, GATA6-AS1, and DICER1-AS1 in cervical cancer tissues and normal tissues, respectively. *P < 0.05.

Based on the above analysis and literature reports, lncRNAs with significant differences were selected for verification. As shown in Table 4, lncRNAs such as CDKN2B-AS1 (Gui and Cao, 2020; Zhang et al., 2018), MIR205HG (Dong et al., 2019; Li et al., 2019; Yin et al., 2022), HAGLROS (Li et al., 2021; Chen et al., 2018a), GATA6-AS1 (Gong et al., 2020; Wang et al., 2020) and DICER1-AS1 (Li et al., 2023) are related to cancer research. Therefore, these lncRNAs were selected for verification. The expression levels of these five lncRNAs in 306 cervical cancer tissues were compared with those in 13 normal tissues utilizing the GEPIA database. The results showed that CDKN2B-AS1, MIR205HG, and HAGLROS were highly expressed in cervical cancer (Figures 5A–C), whereas GATA6-AS1 and DICER1-AS1 showed low expression levels in cervical cancer (Figures 5D,E). Notably, the expression of DICER1-AS1 was not statistically significant (Figure 5E).

**TABLE 4:** Research target of differentially expressed lncRNAs in various diseases.

LncRNA	Up or down	Research target	Disease	References
CDKN2B-AS1	Up	CDKN2B-AS1/miR-4458/MAP3K3	Osteosarcoma	Gui and Cao (2020)
		ANRIL(CDKN2B-AS1)/miR-125a-3p/FGFR1/MAPK	Head and neck squamous cell carcinoma	Zhang et al. (2018)
MIR205HG	Up	MIR205HG/SRSF1/KRT17	Cervical cancer	Dong et al. (2019)
		MIR205HG-miR-122-5p/FOXP2	Cervical cancer	Li et al. (2019)
		LncRNA miR205HG/HNRNPA0	Cervical cancer	Yin et al. (2022)
HAGLROS	Up	HAGLROS-miR-100/SMARCA5	Non-small cell lung cancer	Li et al. (2021)
		STAT3/lncRNA HAGLROS/mTOR	Gastric cancer	Chen et al. (2018a)
GATA6-AS1	Down	GATA6-AS1/miR-543/RKIP	Non-small cell lung cancer	Gong et al. (2020)
		GATA6-AS1/miR-324-5p/FBXO11/SP1	Lung cancer	Wang et al. (2020)
DICER1-AS1	Down	DICER1-AS1/miR-650/MAPK/ERK	Colorectal cancer	Li et al. (2023)

#### 3.5.2 KM-Plotter predicts survival curves for differentially expressed lncRNAs

**FIGURE 6:**
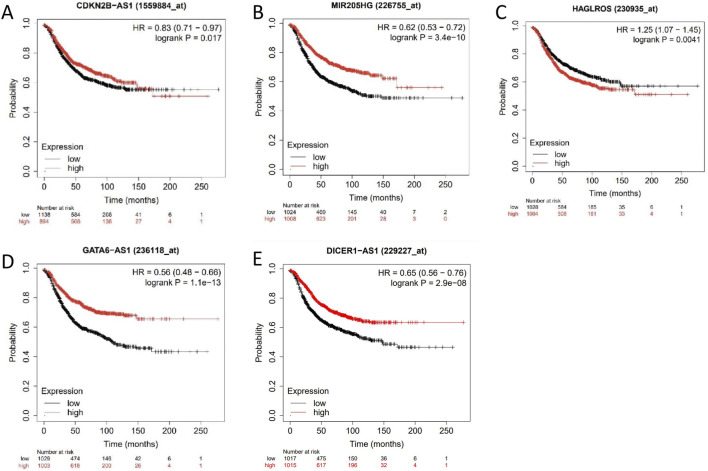
Survival curve analysis of differentially expressed lncRNAs. **(A–E)** Survival curves of patients with cervical cancer with lncRNA CDKN2B-AS1, MIR205HG, HAGLROS, GATA6-AS1, and DICER1-AS1 in the KM-Plotter database.

The prognostic significance of CDKN2B-AS1, MIR205HG, HAGLROS, GATA6-AS1, and DICER1-AS1 in cervical cancer patients was evaluated by using the KM-Plotter database. The analysis showed that high expression levels of CDKN2B-AS1, MIR205HG, and HAGLROS were associated with a poorer prognosis (Figures 6A–C). In contrast, low expression levels of GATA6-AS1 and DICER1-AS1 were also connected to a poorer prognosis (Figures 6D,E).

#### 3.5.3 Validation of differentially expressed lncRNAs in normal cervical epithelial cells and cervical cancer cells

**FIGURE 7:**
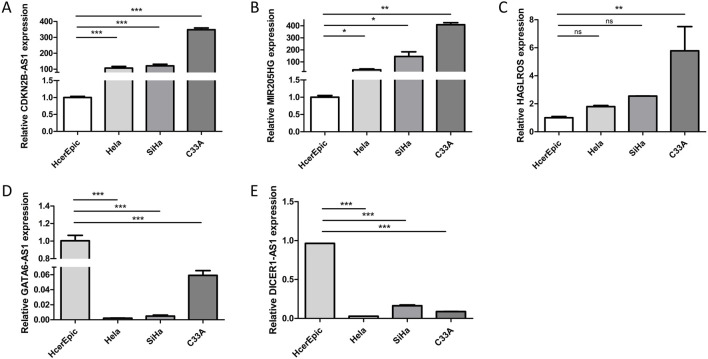
Expression of LncRNA in HcerEpic cells and cervical cancer cells. **(A–E)** RT-qPCR was performed to detect the relative expression of CDKN2B-AS1, MIR205HG, HAGLROS, GATA6-AS1, and DICER1-AS1 in HcerEpic, Hela, SiHa, and C33A cells. *P < 0.05, **P < 0.01, ***P < 0.001.

To further validate the microarray results of differentially expressed lncRNAs, we employed RT-qPCR to detect the transcripts of the five aforementioned lncRNAs in HcerEpic and HeLa, SiHa, and C33A cells. The results demonstrated that the expression levels of CDKN2B-AS1, MIR205HG, and HAGLROS were significantly higher in HeLa, SiHa, and C33A cells than in HcerEpic (Figures 7A–C). Conversely, GATA6-AS1 and DICER1-AS1 showed lower expression levels in the cancer cell lines (Figures 7D,E), and these differences were statistically significant (*P* < 0.05).

#### 3.5.4 Differential expression of lncRNA in HcerEpic cells overexpressing HPV16 E6/E7

**FIGURE 8:**
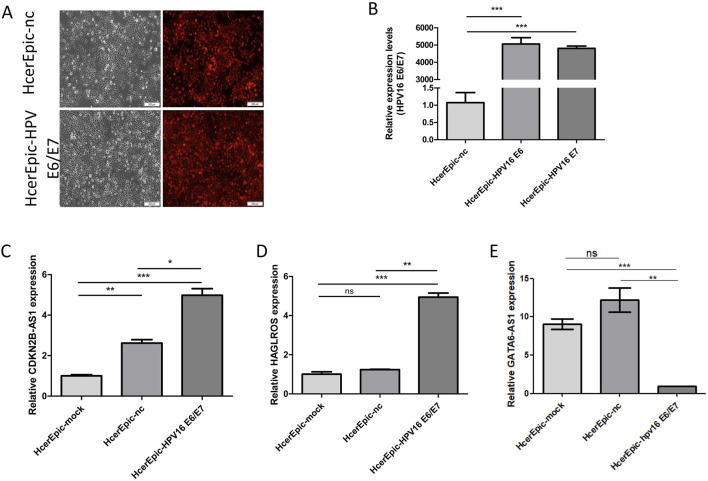
Differential expression of lncRNAs in HcerEpic cells overexpressing HPV16 E6/E7. **(A)** Fluorescence microscopy of HcerEpic cells transfected with lentivirus overexpressing HPV16 E6/E7 to observe the infection efficiency. **(B)** RT-qPCR to detect the transcription of HPV16 E6/E7 mRNA. **(C–E)** RT-qPCR to detect CDKN2B-AS1 in HcerEipc cells overexpressing HPV16 E6/E7, relative expression of HAGLROS and GATA6-AS1. *P < 0.05, **P < 0.01, ***P < 0.001.

To verify whether differentially expressed lncRNAs were associated with HPV16 infection, HcerEpic cells were infected with recombinant lentivirus CV224-HPV16 E6/E7 to establish a model cells overexpressing HPV16 E6/E7. The transfection efficiency was assessed by fluorescence microscopy (Figure 8A). RT-qPCR was employed to detect the transcription levels of HPV16 E6 and E7 in the model cells (Figure 8B). The expression levels of CDKN2B-AS1, MIR205HG, HAGLROS, GATA6-AS1, and DICER1-AS1 in the model cells were further evaluated. The results showed that the expression of CDKN2B-AS1 and HAGLROS was significantly increased, while the expression of GATA6-AS1 was significantly decreased (Figures 8C–E), and the differences were statistically significant (*P* < 0.05). These findings imply that CDKN2B-AS1, HAGLROS, and GATA6-AS1 might be regulated by HPV16 E6/E7 in human normal cervical epithelial cells.

## 4 Discussion

With the emergence of high-throughput technologies, a substantial number of dysregulated lncRNAs have been discovered in cervical cancer (Bai et al., 2023; Liu et al., 2023). These lncRNAs have been increasingly regarded as signature molecules of cervical cancer, and have drawn extensive attention. Notable examples include H19 (Dai et al., 2023; Lee et al., 2003), HOTAIR (Sun et al., 2021), and MALAT1 (Hao et al., 2020). These lncRNAs show high expression levels in cervical cancer tissues and are associated with the proliferation and migration of cervical cancer cells. Chronic infection with high-risk HPV subtypes is closely related to the development of cervical cancer. E6 and E7 are identified as key “HPV carcinogens” that drive the progression of cervical cancer (Fan and Shen, 2018). The dysregulated lncRNAs and their associated regulatory pathways in cervical cancer may serve as potential molecular targets for its diagnosis and treatment.

In this study, we analyzed the differential expression profiles of lncRNA and mRNA in cervical cancer patients via high-throughput RNA sequencing The results showed that there were 7,991 differentially expressed lncRNAs in cervical cancer tissues, with 3,608 being upregulated and 4,383 downregulated (Fold change >2 and *P* < 0.05). Additionally, 5,886 mRNAs were also significantly differentially expressed, including 3,666 that were upregulated and 2,220 that were downregulated. Interaction analysis of the proteins encoded by these significantly different mRNAs demonstrated a strong interaction among the proteins CTNNB1, ATXN2, PRKCD, FCGR3B, UQCRH, RPS28, and KIF15.

The BP, CC, and MF were analyzed by GO enrichment analysis as follows: Upregulated mRNAs are primarily involved in cellular molecular metabolic processes, the cell cycle, organelle organization, growth and development, and other biological processes. These mRNAs are mostly associated with cytoplasmic and organelle components and display molecular functions such as nucleic acid binding, protein binding, and catalytic activity. Conversely, the downregulated mRNAs participate in multicellular biological processes, may have bioadhesion functions, and are connected to myofibrils, the extracellular matrix, and plasma membrane components. Their molecular functions include protein binding and metal ion binding, among others. KEGG pathway enrichment analysis showed that the upregulated mRNAs may be involved in the regulation of the cell cycle, splicing body, and cellular senescence, and are associated with Alzheimer’s disease, the Fanconi anemia pathway, and Parkinson’s disease. The downregulated mRNAs are primarily involved in the regulation of the cell cycle, spliceosome activity, and cellular aging. Additionally, these downregulated mRNAs are associated with insulin and bile secretion and are connected to signaling pathways such as cAMP and MAPK, which may regulate neuroactive ligand-receptor interactions and other pathways. Moreover, analysis of the 20 antisense lncRNAs with the most significant changes revealed that these lncRNAs might exert their biological functions by influencing the corresponding mRNAs, indicating their potential as marker molecules for studying the progression of cervical cancer.

To validate the results of the microarray analysis, we utilized a combination of predictions from the GEPIA database and RT-qPCR assays. We identified the upregulated lncRNAs CDKN2B-AS1, MIR205HG, and HAGLROS, which showed significant differences from the microarray results, as well as the downregulated lncRNAs GATA6-AS1 and DICER1-AS1. The results of GEPIA database showed that CDKN2B-AS1, MIR205HG, and HAGLROS were highly expressed in cervical cancer, and patients with high expression levels had poorer prognoses. Conversely, GATA6-AS1 and DICER1-AS1 were found to be lowly expressed in cervical cancer, and patients with low expression levels had poorer prognoses. We measured the relative expression of these five lncRNAs in HcerEpic and cervical cancer cell lines using RT-qPCR. The results were consistent with the predictions from the GEPIA database, and the differences were statistically significant (*P* < 0.05). These findings show that the observed trends are in line with the microarray results, thereby confirming the reliability of the lncRNA microarray data.

HPV infection is a major risk factor for cervical cancer. In a study, E6 and E7 oncoproteins were found to be strongly associated with cell adhesion in HPV-induced immunosuppression (Dai et al., 2022). In addition to cervical cancer, HPV16 E6/E7 has been associated with head and neck squamous cell carcinoma (HNSCC), e.g., HPV-positive and HPV-negative HNSCC require different B-cell-centered targeting strategies to induce antigen-specific intratumor maturation of TLS and driver B cells (Ruffin et al., 2023). HPV is also capable of preventing a strong immune response in infected cells by reducing chemokine expression, among other mechanisms (Attademo et al., 2020). In this study, to investigate the relationship between differentially expressed lncRNAs and high-risk HPV infection, we constructed a cell line stably overexpressing HPV16 E6/E7 by transfecting HcerEpic cells with the recombinant lentiviral vector CV224-HPV16 E6/E7. The results of the RT-qPCR assay showed that in HcerEpic cells overexpressing HPV16 E6/E7, the expression levels of CDKN2B-AS1 and HAGLROS were significantly increased, while the expression of GATA6-AS1 was decreased. These findings imply that CDKN2B-AS1, HAGLROS, and GATA6-AS1 may be regulated by HPV16 E6/E7 in human normal cervical epithelial cells.

Although the biological functions of microarray-screened lncRNAs have been studied in cervical cancer, such as the knockdown of MIR205HG, which significantly reduced the proliferation, migration, and invasive ability of cervical cancer cells (Yin et al., 2022), and the oncogenic role of LINC000511 in regulating the expression of PGK1 (Xin et al., 2023), the vast majority of differentially expressed lncRNAs are still poorly understood in terms of their roles in cervical cancer. In this study, we screened a large number of dysregulated lncRNAs in cervical cancer using high-throughput sequencing and confirmed our findings through a combination of GEPIA and RT-qPCR. However, this study did not explore how HPV16 E6/E7 regulates these lncRNAs and their regulatory mechanisms on cervical cancer cells. We plan to continue to supplement the regulatory experiments of HPV16 E6/E7 on lncRNAs and the functional experiments of lncRNAs to provide evidence for their impact on the molecular mechanisms of cervical cancer.

The rapid development of machine learning (ML) methods in recent years has revolutionized the research on the association of long chain non-coding RNAs (lncRNAs) with human diseases. Compared with the time-consuming and labor-intensive characteristics of traditional experimental methods, machine learning-based methods achieve high-throughput prediction and prioritization of disease-associated lncRNAs by integrating multi-omics data. For example, Guo et al. (2022) proposed a machine learning-based model called LDACE to predict potential lncRNA-disease associations by combining extreme learning machine (ELM) and convolutional neural network (CNN). Similarly, Chen et al. (2018b) proposed a novel framework called IMCMDA for inferring potential miRNA-disease associations. The powerful advantage of machine learning techniques in revealing lncRNA and miRNA disease regulatory mechanisms. Yi et al. (2020) developed a stacked integrated computational framework, RPI-SE, for efficient prediction of ncRNA-protein interactions. Meanwhile, k-mer sparse matrices were used to extract effective features of ncRNA sequences. In addition, Bao et al. (2019) who provided LncRNADisease 2.0 to organize the study of lncRNA-disease association, which provided new ideas for potential clinical applications related to lncRNA. Whereas this study relies on traditional methods of bioinformatics analysis and lacks machine learning methods to advance the prediction of lncRNA-disease associations, we will incorporate computational methods of machine learning to enhance the exploration of cutting-edge research in the next study.

In conclusion, this study identified the lncRNAs related to cervical cancer, along with their associated biological processes, molecular functions, and molecular biological pathways. This work lays a molecular foundation for predicting biomarkers for cervical cancer and provides bioinformatic data to support its treatment.

## Data Availability

The raw data supporting the conclusions of this article will be made available by the authors, without undue reservation.
