# Supplementary material for: Bioinformatics analysis of lncRNA and mRNA differentially expressed in patients with cervical cancer
Source: Front Bioinform. 2025 Aug 1;5:1605681. doi: 10.3389/fbinf.2025.1605681 (PMC12354555; doi:10.3389/fbinf.2025.1605681)
Supplement: Supplementary file 1 [file Supplementaryfile1.docx]

**Supplementary drawing**

1. mRNA differential expression


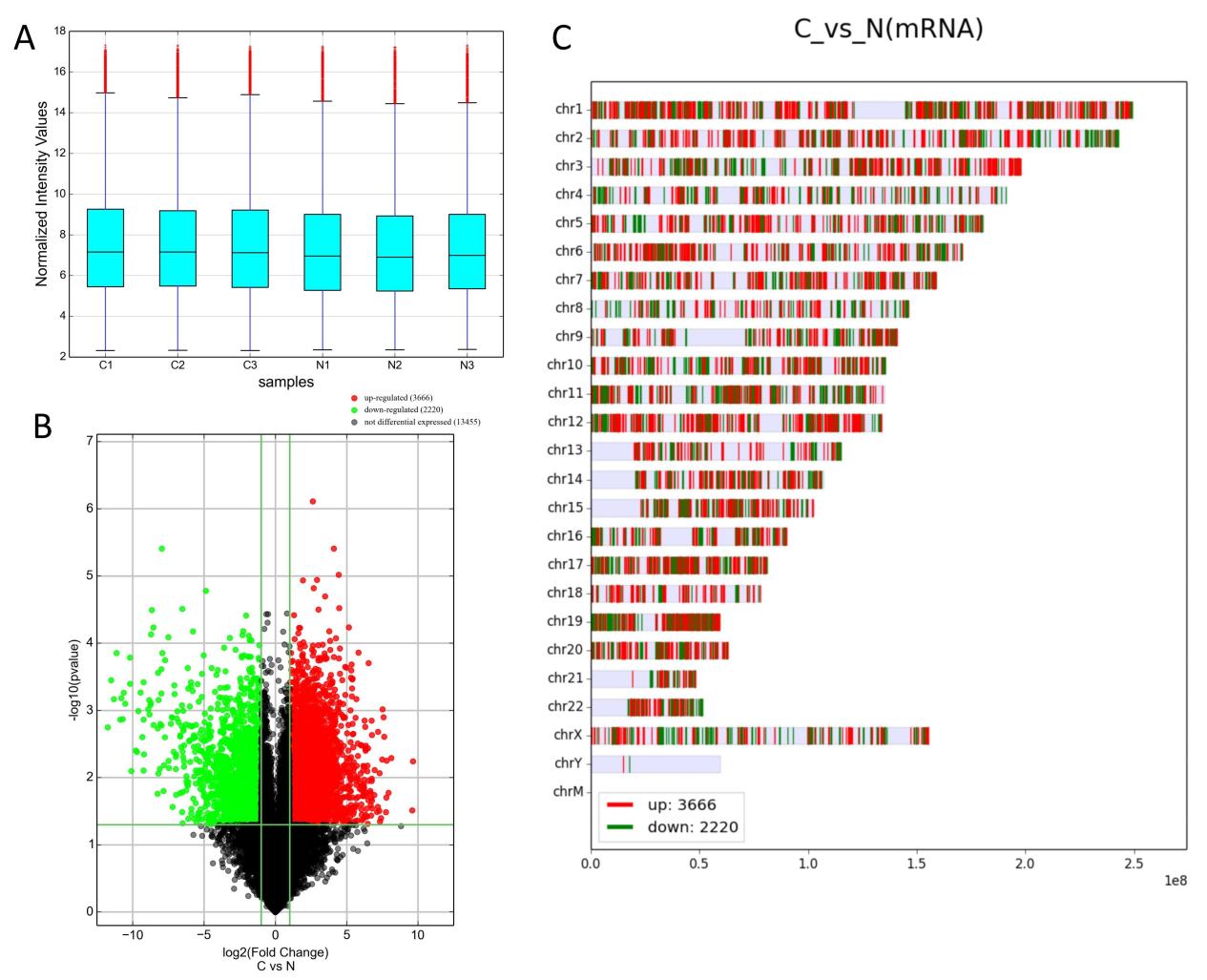


**Figure S1 Differentially expressed mRNAs in cervical cancer tissues and paracancerous tissues**

A: box plot of the dispersion of the six data sets of mRNAs. B: volcano plot of differentially expressed mRNAs showing up-regulated 2-fold mRNAs in red and down-regulated 2-fold mRNAs in green. C:Localization of up-regulated and down-regulated mRNAs in chromosomes.

**Table S1 Differently expressed mRNAs**

| Up-regulated mRNAs | |  | Down-regulated mRNAs | |
| --- | --- | --- | --- | --- |
| mRNA | Fold-change |  | mRNA | Fold-change |
| TEX101 | 799.6322232 |  | EDC4 | 3418.028079 |
| FOXA1 | 771.9272086 |  | SPACA7 | 2896.371611 |
| IVL | 279.0135803 |  | EIF3L | 2544.810592 |
| SERPINB4 | 244.1780375 |  | CAPNS1 | 2231.940986 |
| SHISA2 | 223.0965098 |  | PRDM14 | 1868.574021 |
| SERPINB3 | 219.259605 |  | OR2A12 | 1774.630147 |
| KRT6A | 215.4854726 |  | NR4A1 | 1649.659776 |
| EMC6 | 197.9421303 |  | CHRNA9 | 1575.509313 |
| HOXC13 | 191.3552138 |  | SLC25A3 | 1462.545102 |
| SLC28A3 | 182.7079416 |  | CDC25B | 1164.604971 |
| APOBEC3B | 179.3878538 |  | TRPM4 | 1155.397583 |
| IGFL1 | 169.385167 |  | NOD2 | 1089.938285 |
| EN1 | 161.0694901 |  | GATA1 | 879.5432228 |
| PRC1 | 158.0278547 |  | DNER | 825.9174221 |
| NMU | 155.6494138 |  | NSUN5 | 773.0679407 |
| C15orf48 | 146.5090391 |  | NHLRC4 | 695.5086272 |
| KIF4B | 144.7202037 |  | MYH11 | 638.6944335 |
| TOP2A | 139.1320163 |  | SUPV3L1 | 600.6177912 |
| RARRES1 | 124.33118 |  | SLC25A28 | 600.2787138 |
| E2F8 | 121.8580796 |  | GFRA1 | 599.9244695 |
